# Evaluation of Chitosan-based Dressings in a Swine Model of Artery-Injury-Related Shock

**DOI:** 10.1038/s41598-019-51208-7

**Published:** 2019-10-10

**Authors:** Yao-Horng Wang, Chuan-Chieh Liu, Juin-Hong Cherng, Gang-Yi Fan, Yi-Wen Wang, Shu-Jen Chang, Zhi-Jie Hong, Yung-Chang Lin, Sheng-Der Hsu

**Affiliations:** 1 Department of Veterinary Medicine, National Chung Hsing University, Taichung, Taiwan, R.O.C.; 2 Department of Nursing, Yuanpei University of Medical Technology, Hsinchu, Taiwan, R.O.C.; 3 Department of Cardiology, Cardinal Tien Hospital, Taipei, Taiwan, R.O.C.; 4 Department and Graduate Institute of Biology and Anatomy, National Defense Medical Center, Taipei, Taiwan, R.O.C.; 5 Department of Gerontological Health Care, National Taipei University of Nursing and Health Sciences, Taipei, Taiwan, R.O.C.; 6 Graduate Institute of Medical Sciences, National Defense Medical Center, Taipei, Taiwan, R.O.C.; 7 Division of Rheumatology/Immunology/Allergy, Department of Internal Medicine, Tri-Service General Hospital, National Defense Medical Center, Taipei, Taiwan, R.O.C.; 8 Division of Traumatology, Department of Surgery, Tri-Service General Hospital, National Defense Medical Center, Taipei, Taiwan, R.O.C.

**Keywords:** Molecular biophysics, Thrombosis, Circulation

## Abstract

Uncontrolled haemorrhage shock is the highest treatment priority for military trauma surgeons. Injuries to the torso area remain the greatest treatment challenge, since external dressings and compression cannot be used here. Bleeding control strategies may thus offer more effective haemostatic management in these cases. Chitosan, a linear polysaccharide derived from chitin, has been considered as an ideal material for bleeding arrest. This study evaluated the potential of chitosan-based dressings relative to commercial gauze to minimise femoral artery haemorrhage in a swine model. Stable haemostasis was achieved in animals treated with chitosan fibre (CF) or chitosan sponge (CS), resulting in stabilisation of mean arterial pressure and a substantially higher survival rate (100% vs. 0% for gauze). Pigs receiving treatment with CF or CS dressings achieved haemostasis within 3.25 ± 1.26 or 2.67 ± 0.58 min, respectively, significantly more rapidly than with commercial gauze (>100 min). Moreover, the survival of animals treated with chitosan-based dressings was dramatically prolonged (>180 min) relative to controls (60.92 ± 0.69 min). In summary, chitosan-based dressings may be suitable first-line treatments for uncontrolled haemorrhage on the battlefield, and require further investigation into their use as alternatives to traditional dressings in prehospital emergency care.

## Introduction

Moderate to severe battlefield injury and haemorrhage is the highest treatment priority for military trauma surgeons and researchers. Traumatic haemorrhage of military personnel injured in the line of duty accounts for more than 40% of pre-hospitalisation deaths within the first 24 hours of injury^1^. In addition, uncontrolled haemorrhage is the second leading cause of death in civilian trauma patients^1–4^. Life-threatening coagulopathy is frequently observed in severe trauma patients. Trauma-associated coagulopathy is caused by metabolic acidosis, hypothermia, and dysfunctional clot formation. Moreover, immediate complications such as multiple organ failure may result from prolonged hypotension, sepsis, and plasma product transfusion^5–8^. According to statistics from a previous study, approximately 24% of deaths resulting from combat could have been prevented with immediate and effective treatment^9^. Most of these victims died because of preventable compressible or non-compressible haemorrhaging. Therefore, it is critical to develop a first-line measure to control haemorrhage.

Since 2003, several haemostatic products have been developed to treat compressible haemorrhages arising from battlefield injuries. However, 15% of battlefield injuries occur to the torso area (chest, abdomen, pelvis, and back), where external dressings and compression are not useful^10,11^. Haemorrhage control seems to be a promising approach to avoiding this problem, so studying bleeding control may suggest effective haemostatic methods that could save lives during the acute emergency phase. The haemostatic efficacy and acute safety of haemostatic dressings have been tested in swine on groin arterial haemorrhages that could not be controlled with standard surgical cotton gauze. These dressings are most promising, since they allow precise application even under extremely cold temperatures or insufficient light conditions.

Currently, there are more than 10 commercially available chitosan-based dressings that are utilised in the acute emergency phase. Chitosan haemostatic dressings are superior to traditional granular/powder agents due to their effective bleeding arrest, ease of use, and precise application to deep, penetrating wounds, as well as possible antibacterial properties and regeneration-stimulating effects^12^. In this context, we tested two novel chitosan-based dressings, chitosan fibre (CF) and chitosan sponge (CS), for use as first aid measures in a swine model of standard arterial haemorrhage. These dressings are made from poly-D-glucosamine and poly-N-acetyl glucosamine derived from natural organic chitosan. Chitosan is a linear amino polysaccharide produced by deacetylation of naturally occurring chitin and exhibits interesting biological, physiological, and pharmacological properties. Chitosan is rich in glycosaminoglycan, which participates in cell–cell and cell–matrix interactions, cell proliferation and migration, and in cytokine and growth factor signalling, thus locally modulating biologic activities^13^. Major bioactivities include the promotion of wound healing, haemostatic activity, immunity enhancement, hypolipidemic activity, mucoadhesion, and antimicrobial activity. Despite its low water solubility, chitosan has become a common substance in biomaterials research and has been used for several applications in the biomedical field.

The soft, nonwoven CF dressing is made of chitosan fibres that can accelerate haemostasis and wound care during arterial haemorrhage. With its unique flexibility, the CF dressing is suitable for various wound surfaces, regardless of location, size, sinus, or depth. Chitosan fibre manufacturing techniques have been established during the past two decades. Generally, CF dressing is prepared using a wet-spinning process that produces fibres by dissolving the polymer in a solvent (e.g. acetic acid), after which the polymer solution is extruded via dies into a nonsolvent (e.g. aqueous sodium hydroxide). The polymer precipitate emerges in the form of fibres that can be washed, drained, and dried. The CF dressing uses the properties of chitosan fibres to combine an adequate porous structure with excellent degradability and mechanical properties.

The CS dressing, which is a light-yellow sponge, is made of natural organic chitosan particles and can be comfortably attached to the skin to seal a wound. Generally, CS dressing is prepared using a freeze-drying method, resulting in a sponge-like form. It keeps the wound sterile for as long as seven days. Additionally, the porosity of the CS dressing allows rapid absorption of a large volume of exudate from the injury site. Following absorption, the exudate interacts with the CS dressing to form a moist environment covering the wound for up to seven days.

This study aims to compare the efficacy of chitosan-based dressings and commercial gauze in a swine model. We explored the potential of these two chitosan-based dressings to minimise femoral artery haemorrhage and achieve immediate haemostasis in a swine model. Based on our results, chitosan-based dressings require further study as alternatives to traditional dressings in prehospital emergency care.

## Results

### Background physiological and haematological data

Ten animals were randomly assigned to one of three treatment groups. We collected blood samples for physiological analysis. The pre-experiment baseline physiological and haematological measurements are presented in Table 1. There were no significant differences in baseline measurements among the groups.

**Table 1:** Baseline physiological and haematological measurements of dressings used for pigs.

Outcome	CF (N = 4)	CS (N = 3)	Gauze (N = 3)	Overall *p*
HGB (g/dL)	16.40 ± 0.51	16.30 ± 0.87	16.33 ± 0.91	0.984
HCT (%)	58.18 ± 4.96	55.33 ± 6.05	55.50 ± 6.48	0.766
PLT (1,000/μL)	672.00 ± 67.56	687.00 ± 171.43	801.67 ± 18.01	0.278
pH	7.38 ± 0.10	7.39 ± 0.06	7.29 ± 0.11	0.363
HCO_3 (mM)_	21.68 ± 2.17	22.70 ± 2.03	16.53 ± 8.78	0.323

Data expressed as mean ± SE and analysed by one-way ANOVA. NS = *p* > 0.1.

ANOVA, analysis of variance; HGB, haemoglobin; PLT, platelets; NS, not significant.

### Haemostatic achievement

**Figure 1:**
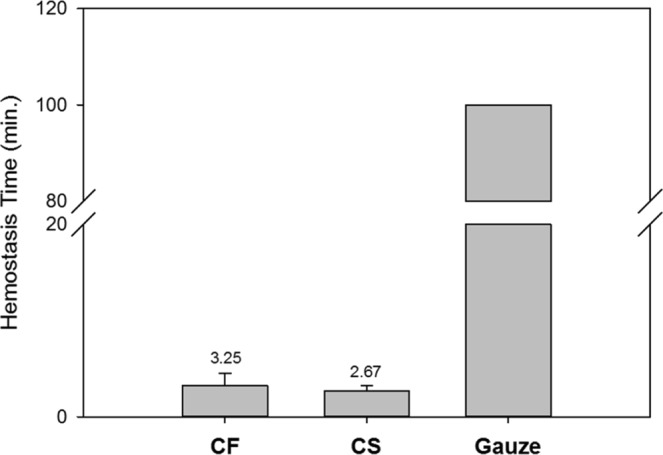
The haemostatic outcomes of CF and CS dressings compared to commercial gauze application in a swine model of arterial haemorrhage. Median time to haemostasis. Error bars represent the 95% confidence interval of the median. The chitosan-based dressings promoted haemostasis significantly faster than commercial gauze.

To compare the haemostatic effectiveness of CF, CS, and commercial gauze, the time required to achieve haemostasis was calculated from the initial application of the dressing. Initial haemostasis was considered to have occurred when the bleeding had stopped for at least 3 min after compression. The mean time to haemostasis and other haemostatic outcomes are shown in Table 2. Stable haemostasis was achieved with both the CF and the CS dressings, resulting in stabilisation of MAP and a significant improvement in survival, with 100% of animals surviving throughout the experiment. In contrast, 0% of the gauze-treated animals survived for the entire experiment. The pigs treated with CF and CS dressings achieved haemostasis within 3.25 ± 1.26 and 2.67 ± 0.58 min, respectively, which was significantly less than the time to haemostasis with commercial gauze (>100 min; Fig. 1). The quantity of fluid required for the resuscitation of the CF- and CS-treated animals was 30.55 and 31.58 mL/kg, respectively, which was significantly less than for the gauze-treated pigs (188.32 mL/kg; Table 2). When the stability of the haemostasis provided by the CF and CS dressings was examined under simulated walking, there was no recurrence of bleeding in the animals.

**Table 2:** Outcomes of treating a groin arterial haemorrhage.

Outcome	CF (N = 4)	CS (N = 3)	Gauze (N = 3)	Overall *p*
Total time until bleeding stopped (min)	3.25 ± 1.26	2.67 ± 0.58	>20	NS
Total resuscitation fluid (mL/kg)	30.55	31.58	188.32	<0.001
Survival rate (%)	100%	100%	0%	NS
Survival time (min)	>180	>180	60.92 ± 0.69	NS

Data expressed as mean ± SD and analysed by one-way ANOVA. NS = *p* > 0.1.

ANOVA, analysis of variance; NS, not significant.

**Figure 2:**
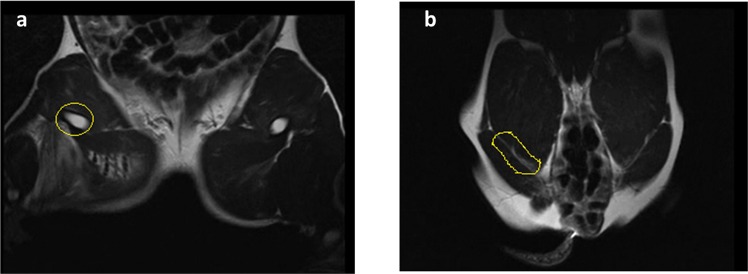
Representative MRI of the arterial circulation in the hind legs of surviving animals treated with CF and CS dressings 3 hours after surgery (**a**,**b**). Note that the blood flow in the treated femoral artery was not affected (yellow circle).

Representative MRI images of the treated pigs showed no significant increase in wound temperature after the application of the haemostatic dressings (data not shown). A complete blockage of blood flow was observed in the femoral artery at the wound site in all surviving animals (Fig. 2). Despite this, blood flow through collateral arteries was not significantly affected.

### Survival

**Figure 3:**
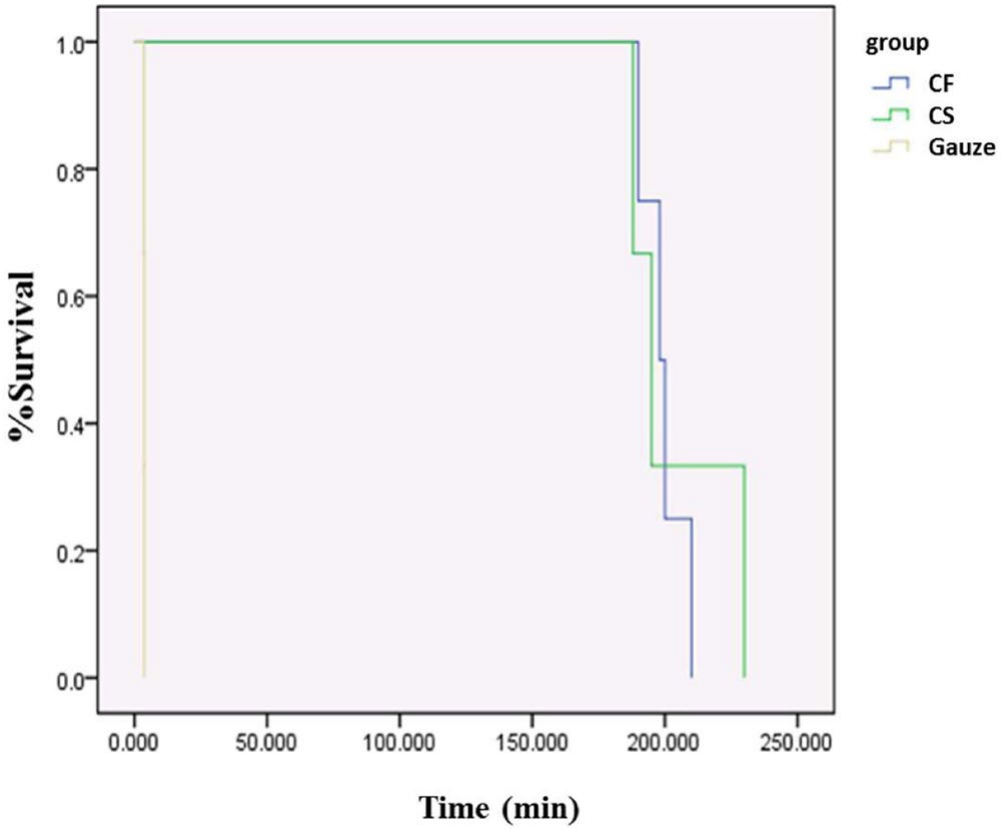
Kaplan-Meier analysis. The chitosan-based dressings supported the survival of animals for a significantly longer period than commercial gauze.

All of the CF- and CS-treated animals survived for at least 180 min after the procedure, significantly longer than those treated with commercial gauze (60.92 ± 0.69 min; Fig. 3), which died by bleeding to death. The average bleeding times for all dressing types are given in Table 2. Notably, there were no differences in survival rate or time between the two chitosan-based dressing treatments.

## Discussion

The present study shows that the CF and CS dressings achieved haemostasis effectively, rapidly controlling moderate-to-severe haemorrhage, which represented a great improvement over commercial gauze. The reduced bleeding time resulted in less blood loss and a lower fluid resuscitation rate. It is reasonable to postulate that the large blood loss with commercial gauze, and the subsequent high fluid resuscitation rate, reduced the clotting capacity of the blood, leading to unfavourable outcomes. Both chitosan-based dressings exhibited similar efficacy in this haemorrhage model. Previous studies have also shown that CS dressings effectively achieve haemostasis, even in the presence of anticoagulants in the blood. Therefore, we hypothesise that interactions between the positively charged ions in the chitosan and the negatively charged ions in red blood cell membranes may enhance haemagglutination and encourage platelet adhesion and activation via protein adsorption and orientation, thus affecting cellular responses to proteins^14,15^. It has also been demonstrated that the porosity of this dressing facilitates higher ventilation over the injury site, allowing more oxygen to diffuse inward across the skin, thereby stimulating the healing process.

Chitosan characteristically carries cations such as -NH_3_^+^, which have a positive effect on haemostasis. If attached to the surface of the chitosan-based dressing, the metal cation–chitosan complex enhances the adhesion of erythrocytes and platelets to the injury site. Chitosan also improves blood coagulation efficiency (either stopping bleeding or controlling haemorrhage). Indeed, it appears to demonstrate better haemostatic efficacy than traditional granular/powder agents, even in the presence of anticoagulants such as heparin. Once the bleeding has stopped, the dressing would then form an antibacterial layer to prevent wound infections and provide an appropriate wound-healing environment. Further, such dressings are compatible with human skin and possess properties that allow ventilation of the wound and absorption of the wound exudate. Previous studies have also demonstrated the safety of chitosan. The biocompatibility and biodegradability of chitosan, combined with its haemostatic and anti-infection activity, make it highly useful and advantageous for use in wound dressings.

The swine model of femoral artery haemorrhage we used in this study represented a severe injury to the groin area with partial destruction of the femoral artery, causing a life-threatening haemorrhage that is impossible to control with commercial gauze, and not manageable using a tourniquet. The strength to this study was that the surgical procedures were performed by a researcher who was blinded to group assignment, which increases confidence in the outcome. Although our approach represents a reliable model for examining the efficacy of haemostatic dressings, there are some limitations of this study that should be addressed in future research. Firstly, considering there are many different types of gauze available for haemostatic management of bleeding, the results in this study can be influenced by the gauze type. Secondly, we are unable to establish a comprehensive comparison between chitosan-based dressings and gauze because of lack of packing time in this study. Although chitosan-based dressings significantly reduced time to haemostasis as compared with control, they require more time for packing according to the procedure. Thirdly, this model does not fully mimic battlefield or accident-induced traumatic injuries. The efficacy of the dressings could be affected by changes in environmental or wound conditions. Lastly, given our small sample size (3 different dressings on 10 animals), this should be followed up with additional studies using a larger sample size.

Currently, the biomedical application of most synthetic polymers is limited by insufficient biocompatibility and poor biodegradability. In contrast, natural polymers such as cellulose, chitin, chitosan, and their derivatives have been proven to be biocompatible and biodegradable. Furthermore, the degradation product of chitosan is nontoxic to humans^16^. Chitosan is also useful as a supporting material for tissue engineering. Previous studies have revealed that both chitosan and chitosan oligomers exhibit antibacterial activity, and that chitosan displays greater antibacterial activity than chitosan oligomers^17,18^. Potential future directions should include conducting chitosan-based dressings testing in more challenging animal models, combining chitosan with other materials to achieve stable haemostasis, and modifying the dressings to facilitate handling to further reduce the packing time.

In summary, a large amount of effort has been invested in the investigation of novel haemostatic products. In this study, a swine model of femoral artery haemorrhage was utilised to evaluate the efficacy of CF and CS dressings. This model mimicked a severe injury to the groin area with partial destruction of the femoral artery, causing a life-threatening haemorrhage that is impossible to control with commercial gauze and not manageable with a tourniquet. The results revealed that both CF and CS dressings reduced the time to haemostasis and the fluid resuscitation rate relative to commercial gauze, and the CF- and CS-treated animals survived significantly longer than the control animals. Additionally, the two types of chitosan dressing were equally efficacious in mitigating blood loss and promoting survival. Based on these findings, CF and CS dressings may be suitable first-line treatments for uncontrolled haemorrhage on the battlefield, and require further investigation into their use as alternatives to traditional dressings in prehospital emergency care.

## Methods

Animal Care Committee at National Defense Medical Center, Taiwan, Republic of China (R.O.C.), approved the procedures involving animal subjects (Institutional Animal Care and Use Committee (IACUC)-15-337). Additionally, all methods were performed in accordance with the relevant guidelines and regulations.

### Experimental dressing

**Figure 4:**
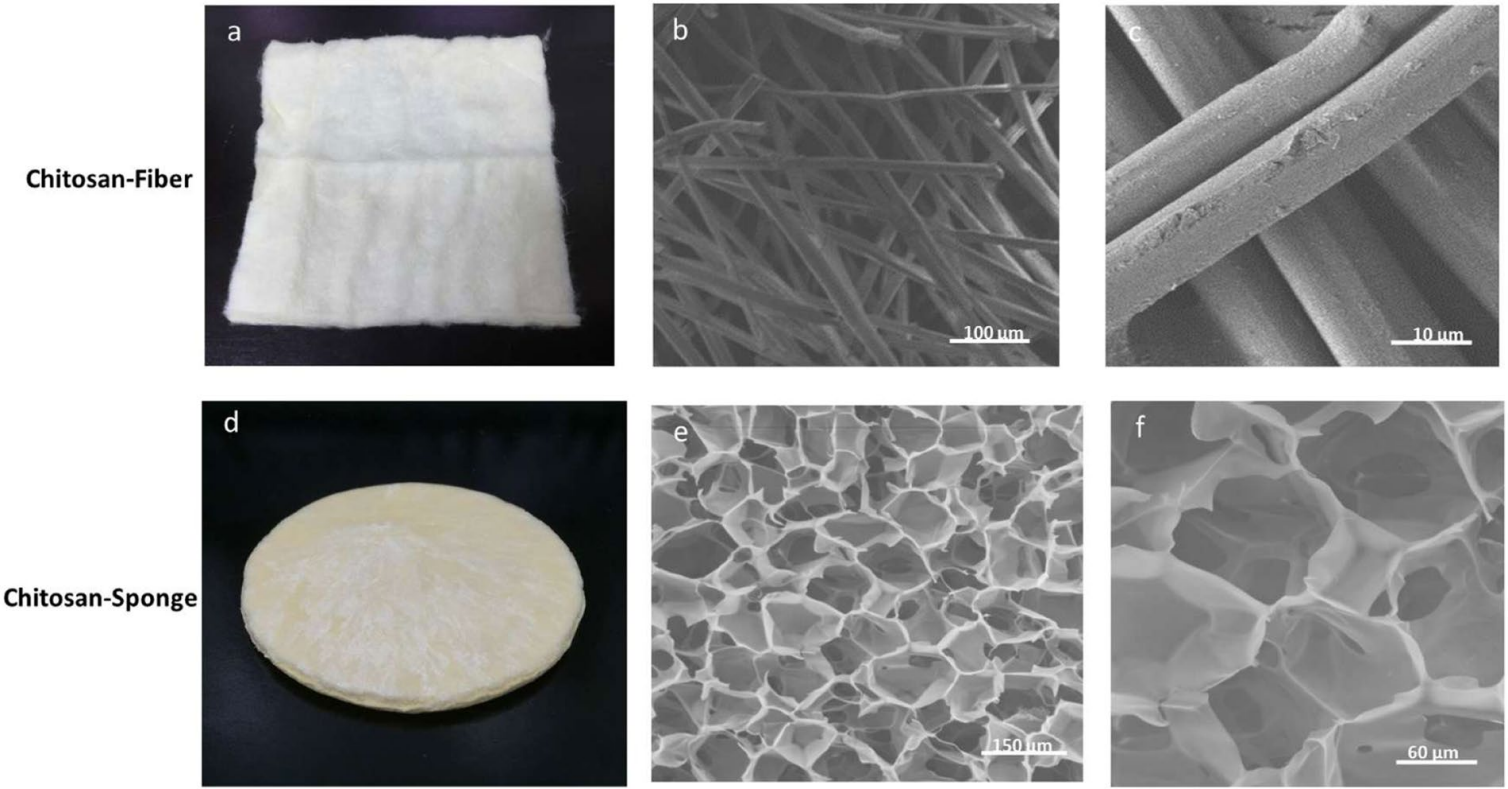
The morphology of chitosan-based dressings. (**a**) Chitosan-fibre (CF) dressing; (**b**) Electron microscopic structure of CF dressing at 100x; (**c**) Electron microscopic structure of CF dressing at 1000x; (**d**) Chitosan-sponge (CS) dressing; (**e**) Electron microscopic structure of CS dressing at 100x; (**f**) Electron microscopic structure of CS dressing at 250x.

Surgical cotton gauze was purchased from China Surgical Dressings Company (R.O.C.) and was applied over the haemostatic dressing to fill the wound cavity. The two novel chitosan-based dressings that were used in the experimental groups were purchased from Coreleader Biotech Corporation (CS dressing; R.O.C.) and the Taiwan Textile Research Institute (CF dressing; R.O.C.). The composite fibres were cut to restrict the crimped fibres or yarns to a staple length between approximately 0.25 and 6.35 cm. The staples were carded to create nonwoven attenuated mats or batts, which were then needle-punched and cropped to a size of 10 × 10 cm. The nonwoven multi-layered chitosan fabric had a base weight of approximately 1.55–31 mg/cm^2^. The morphology of the chitosan-based dressings is shown in Fig. 4.

### *In vivo* methods

We used ten Yorkshire cross-bred pigs (castrated males only) weighing 34–37 kg in this study. The animals were fasted for 12 h prior to surgery, with unlimited access to water. Before surgery, they were injected with buprenorphine (0.025 mg/kg, intramuscular [i.m.]) for analgesia, and glycopyrrolate (0.01 mg/kg, i.m.) to reduce saliva secretion and block bradycardia. The animals were injected with tiletamine-zolazepam (Telazol, 4–6 mg/kg, i.m.) and anesthetised with 5% isoflurane in oxygen via a face mask. The tidal volume and ventilation rate were adjusted to maintain end-tidal carbon dioxide in the range 38–42 mmHg. Anaesthesia was maintained using 1–2% isoflurane in a mixture of oxygen via a ventilator. An intravenous catheter was inserted into the ear vein for venous access, and Lactated Ringer’s solution was maintained at a rate of 5 mL kg^−1^ h^−1^.

### Surgical procedures

**Figure 5:**
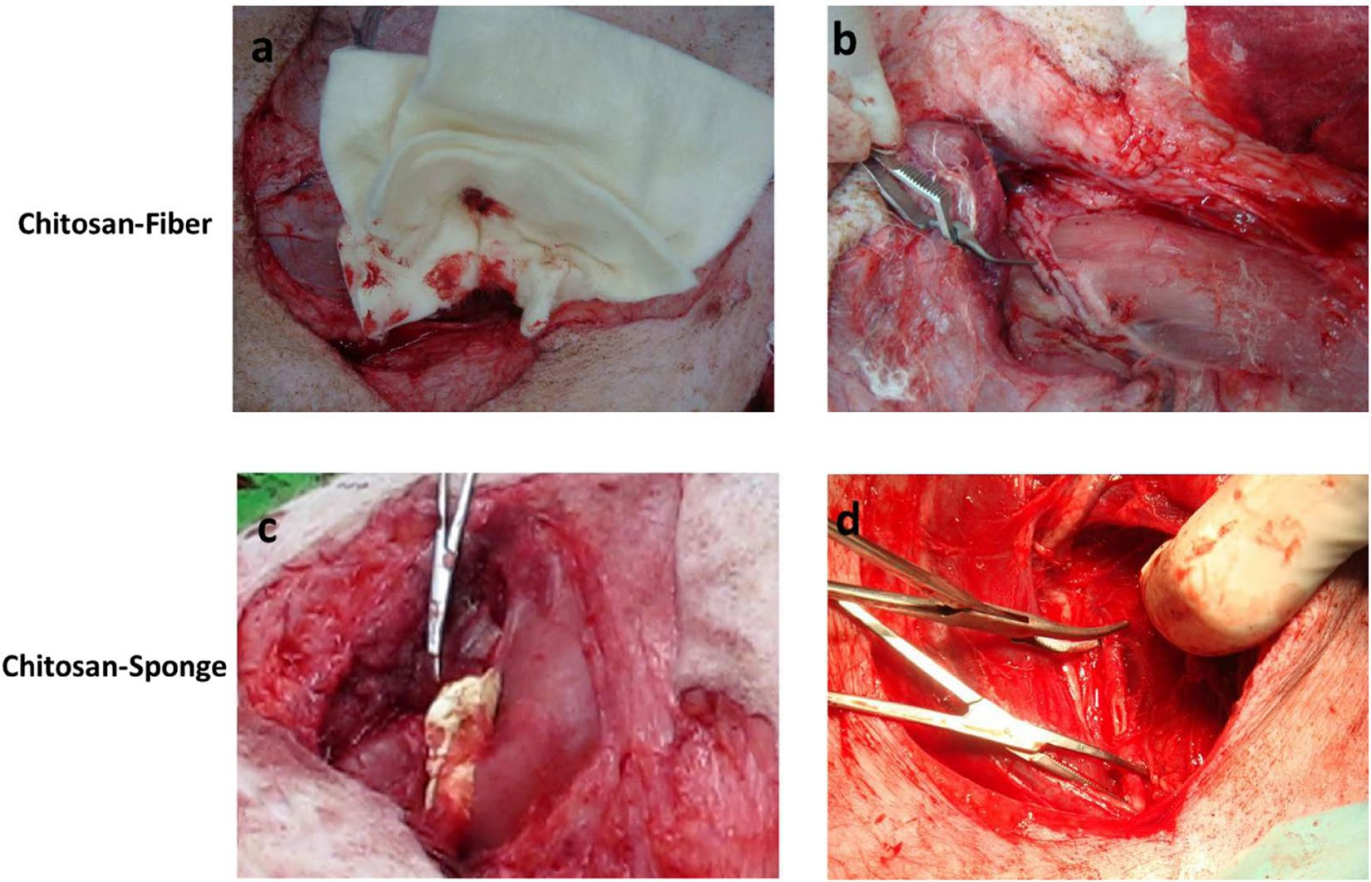
Photographs of preparation of femoral artery haemorrhage in a swine model. (**a**,**c**) groin wound appearance after application of CF and CS dressing; (**b**,**d**) 6-mm-diameter arteriotomy was made on the anterior surface of the vessel using a vascular punch.

The right carotid artery was cannulated for blood collection and the monitoring of blood pressure (systolic, diastolic, and mean) and heart rate throughout the experiment. The femoral artery was identified and partially exposed to approximately 5 cm. The overlying abductor muscle was then removed while taking care to avoid injuring the adjacent femoral vein and nerve. A 2 mL volume of 2% lidocaine was applied to the artery for 5 min to reduce arterial vasospasm and dilate the artery to its normal size. For wound-site temperature monitoring, a microelectrode was sutured to the muscle adjacent to the vessel, but at least 2.5 cm away from the arteriotomy site, to minimise interference with the haemostatic treatment. Baseline mean arterial pressure (MAP) and body temperature were recorded every 10 min during the surgery. A stable MAP of 60 mmHg or higher was required before we proceeded with the rest of the experiment. The artery was occluded proximally and distally with vascular clamps and an arteriotomy 6 mm in diameter was made on the anterior surface of the artery using a vascular punch (International Biophysics, Austin, TX). The vascular clamps were then removed to allow free bleeding for 45 s (Fig. 5).

### Blood sample processing

A sample of 9.0 ml swine blood was collected from a cannulated femoral artery and transferred to tubes containing 1 mL sodium citrate (3.18%). Haemoglobin (HGB), haematocrit (HCT), and platelets (PLT) were tested. Hemocytometric analysis was measured using automated XT-1800 iv (Sysmex Corp., Japan). For the analysis of blood gas (arterial pH and HCO3), blood samples were immediately analysed with Stat Profile® pHOx® (Nova Biomedical, Waltham, MA) analyser.

### Wound treatment and resuscitation

The arterial injury, dressing application, and compression were performed by the same investigator (Hsu, S.D.) for all experiments to minimise variability. The surgeon was blinded to the type of test material to be used for each animal prior to dressing application. The products were applied according to the manufacturers’ instructions. In brief, a package of one of the types of treatment dressing was opened and applied directly over the arterial wound after the 45 s free bleed. After 30 s of compression, a 500 mL volume of Hextend (6% hetastarch in balanced electrolytes plus glucose) was given intravenously for fluid resuscitation to compensate for pre-treatment blood loss. The colloid fluid was administered intravenously at a rate of 100 mL/min to attain a MAP of 65 mmHg (the average baseline blood pressure of anesthetised pigs). After 5 min of compression, haemostasis was observed for 3 min without removal of the laparotomy gauze. If the initial bleeding had not been controlled, the laparotomy gauze and dressing were removed and fresh dressings were applied. Compression was then repeated for 2 min and haemostasis was observed for the next 3 h.

The animals were monitored for up to 3 h or until death, which was pronounced when the end-tidal PCO_2_ and MAP fell below 15 mmHg and 20 mmHg, respectively, and remained at these levels for at least 2 min. The survival time was recorded and the final blood samples (arterial) were collected (for haematological measurements) before the animals were euthanised. The surviving animals were scanned using magnetic resonance imaging (MRI) to obtain images of the arterial blood flow and vascular structures in the legs. In addition, the stability of the haemostasis provided by the haemostatic dressings was tested by flexing and stretching the wounded legs of the surviving animals five times, to mimic walking. At the end of the experiments, the dressings were slowly removed from the wounds to examine the status of the haemostatic clots and patency of the vessels. The animals were then euthanised and tissue samples containing the injured artery, adjacent femoral vein, femoral nerve, and muscle tissues were collected for histologic examination.

### Data analysis

Data were expressed as mean ± standard error of the mean and analysed by one-way Analysis of Variance (ANOVA) test; p < 0.05 was considered statistically significant^19^.

### Ethical approval

Animal Care Committee at National Defence Medical Centre, Taiwan, Republic of China (R.O.C.), approved the procedures involving animal subjects (Institutional Animal Care and Use Committee (IACUC)-15-337).

## Data Availability

The data used to support the findings of this study are available from the corresponding author upon request.

## References

[1] Bellamy RF (1984). Causes of death in conventional warfare: implications for combat casualty care research. Mil. Med..

[2] Oyeniyi BT (2017). Trends in 1029 trauma deaths at a level 1 trauma center. Injury.

[3] Bulger EM (2014). An evidence-based prehospital guideline for external hemorrhage control: American College of Surgeons Committee on Trauma. Prehosp. Emerg. Care.

[4] Gruen RL (2012). Haemorrhage control in severely injured patients. Lancet.

[5] Ratnasekera, A., Reilly, P. & Ferrada, P. Damage control resuscitation in surgical critical care,” In *Damage Control in Trauma Care*, Duchesne, J., Inaba, K., Khan, M., Eds, chapter 15, Springer, AG, (2018).

[6] Maegele M, Spinella PC, Schöchl H (2012). The acute coagulopathy of trauma: mechanisms and tools for risk stratification. Shock.

[7] Brohi K (2008). Acute coagulopathy of trauma: hypoperfusion induces systemic anticoagulation and hyperfibrinolysis. J Trauma.

[8] Kauvar DS, Lefering R, Wade CE (2006). Impact of hemorrhage on trauma outcome: an overview of epidemiology, clinical presentations, and therapeutic considerations. J Trauma.

[9] Kelly JF (2008). Injury severity and causes of death from operation iraqi freedom and operation enduring freedom: 2003–2004 versus 2006. J Trauma.

[10] Sauaia A (1995). Epidemiology of trauma deaths: a reassessment. J Trauma.

[11] Van Oostendorp SE, Tan ECTH, Geeraedts LMG (2016). Prehospital control of life-threatening truncal and junctional haemorrhage is the ultimate challenge in optimizing trauma care; a review of treatment options and their applicability in the civilian trauma setting. Scand J of Trauma, Resusc Emerg Med.

[12] Kheirabadi BS (2009). Comparison of new hemostatic granules/powders with currently deployed hemostatic products in a lethal model of extremity arterial hemorrhage in swine. J Trauma.

[13] Cherng, J. H. The Strategies of Natural Polysaccharide in Wound Healing, Wound Healing - Current Perspectives, K. H. Dogan, IntechOpen, 10.5772/intechopen.80812. Available from: https://www.intechopen.com/books/wound-healing-current-perspectives/the-strategies-of-natural-polysaccharide-in-wound-healing (Nov. 5th 2018).

[14] Stricker-Krongrad AH (2018). Efficacy of chitosan-based dressing for control of bleeding in excisional wounds. ePlasty.

[15] Lord MS, Cheng B, McCarthy SJ, Jung M, Whitelock JM (2011). The modulation of platelet adhesion and activation by chitosan through plasma and extracellular matrix proteins. Biomaterials.

[16] Enescu D, Olteanu CE (2008). Functionalized chitosan and its use in pharmaceutical, biomedical, and biotechnological research. Chem, Eng. Comm..

[17] No HK, Park NY, Lee SH, Meyers SP (2002). Antibacterial activity of chitosans and chitosan oligomers with different molecular weights. Int. J. Food Microbiol..

[18] Jung EJ (2010). Antibacterial activity of chitosans with different degrees of deacetylation and viscosities. Int. J. Food Sci. Tech..

[19] Institute of Laboratory Animal Resource, National Research Council, Guide for the Care and Use of Laboratory Animals, National Academy Press, Washington, D.C. (1996).

